# From Routing to Weighting: Mask-Only Policies for Repairing Incomplete Photovoltaic Monitoring Records

**DOI:** 10.3390/s26206589

**Published:** 2026-10-17

**Authors:** Xianbo Wang, Siqi Zhang

**Affiliations:** 1 Hainan Institute of Zhejiang University, Sanya 572025, China; xbwang@zju.edu.cn; 2 College of Electrical Engineering, Zhejiang University, Hangzhou 310027, China

**Keywords:** condition monitoring, data repair, missing data imputation, photovoltaic monitoring, fault-aware processing, estimator combination, algorithm selection, mixture of experts, multivariate time series

## Abstract

Repairing an incomplete photovoltaic monitoring record requires choosing an imputation operator, and the right choice depends on which evidence the fault left intact. The decision can use only what a monitoring system holds at repair time. We study mask-only policies, which read the observation mask (which cells are missing) and the clock but never the values being repaired, and ask how fine such a decision must be. Two policies are compared: a fault-aware router that diagnoses each missing cell into one of three regimes and dispatches it to one operator, and MAWC, a mask-aware weighted combination that predicts a convex weight over the whole operator pool per cell. Both are fixed on two desert development sites and evaluated once on four held-out sites spanning two further climates and cadences from 1 to 15 min, with mixed fault masks and time-contiguous evaluation segments, two protocol corrections without which a per-cell policy cannot be measured. The router improves on the operator that a deployer could have committed to at every held-out site and lies within 7% of the best fixed assignment of operators to its regimes at three of them. MAWC improves on the router at all four sites, significantly at three, improves on per-regime convex weights at all four, and falls below the regime oracle at two, which no hard router using those regimes can do. Its gain comes from hedging rather than finer selection: a selector trained to pick one operator per cell with the same features loses 15% to 34%. The advantage holds under retrained deep members, doubled replication, changed fault composition and a higher missing rate.

## 1. Introduction

Condition monitoring of a photovoltaic plant rests on a multivariate record that is almost never complete. Inverter faults, sensor failures, logger resets, communication dropouts and scheduled maintenance leave gaps ranging from isolated samples to outages of several days, and because virtually every downstream model assumes a complete input matrix, filling those gaps is an unavoidable stage whose choice propagates into performance assessment, fault diagnosis and forecasting [1,2].

These failure modes do not leave the same traces. A failing sensor blanks one channel while the rest of the station keeps reporting; a logger reset blanks every channel at once; a telemetry glitch removes scattered samples. The families of the repair operator do not consume the same evidence either: interpolation needs the local signal, cross-variable regression needs a contemporaneous channel to survive, and a same-clock-time ensemble only needs the process to repeat daily. A preliminary comparison of operators by the authors on three of the sites used here (unpublished) showed the consequence: the operator that succeeds is determined more by which evidence the fault leaves intact than by the capacity of the model, and the winning family moves along the gap axis from time-local smoothness, through the methods that exploit surviving channels, to periodic repetition. That comparison ranked operators and proposed no rule for choosing among them. Because it is not published, every design choice this paper takes from it is re-derived here from the development sites alone (Appendix B), and none of our conclusions rest on its numbers.

A restriction is shared by this line of work and by the imputation literature more broadly. Studies of missing-data repair evaluate operators, and they evaluate them one missingness pattern at a time. Both choices are natural when the object of study is the operator, and both become binding as soon as the object of study is the rule that chooses among operators. Under a mask that is homogeneous in fault type, every missing cell receives the same diagnosis, so any per-cell rule collapses to a single global choice and its behavior is never exercised. A protocol that reports which operator ranks first also supplies no evidence about how much a pipeline gains by conditioning that choice on the fault at all. The question a monitoring pipeline faces is therefore not addressed by the existing evidence: given that the right operator depends on the fault, how fine does the decision have to be? A pipeline could commit to one operator for all gaps, diagnose a small number of fault types and dispatch, or decide separately for every missing cell, and each step along that scale costs complexity without it being obvious where the returns stop.

The question is sharpened by a constraint that has a specific repair approach and which is easy to overlook. Whatever decides the operator can read only what a monitoring system holds at repair time, which is the observation mask and the clock, never the values being repaired. A selection rule that consults the truth is not a policy but an oracle. The observed part of the record is also available, and the operators themselves consume it, so a policy could, in principle, read the observed context as well. We restrict the policy to the mask deliberately rather than by necessity. A rule that reads only the mask can be fitted on one site and applied at another whose channels, scales and climate differ, because the mask means the same thing everywhere, whereas value-derived features carry site-specific distributions into the rule. This paper therefore studies policies whose entire input is the mask and the calendar, uses oracles only as bounds, and, in Section 4.8, tests what the restriction costs against a policy that also reads observed context.

We compare two such policies at opposite ends of the granularity scale. The first, a fault-aware router, diagnoses each missing cell from the mask into one of three regimes and dispatches it to the operator that the preliminary comparison identified for that regime. Its single free parameter is a duration threshold, specified in physical hours so that the same rule is meaningful at cadences differing by a factor of fifteen. The second, which we call MAWC (mask-aware weighted combination), replaces the hard three-way choice with a weight over the whole operator pool, predicted per cell by a small network from mask-derived and calendar features. Both are calibrated or trained on two desert development sites and evaluated once on four held-out sites spanning two further climates and cadences from 1 to 15 min.

Two reference quantities, both defined in Section 3.1, make the comparison interpretable rather than merely ordinal. The regime oracle is the best fixed assignment of operators to the router’s regimes, chosen with knowledge of the truth. It caps every hard router that uses those regimes, including one whose diagnosis is perfect, so the distance to it separates a deficient rule from a deficient action space. The per-cell oracle caps hard selection at any granularity. Neither bounds a convex combination, since an average of several estimators can lie closer to the truth than any of them, so the two together place the combiner’s gain on a scale rather than reporting it in isolation.

Evaluating a per-cell policy also requires masks that a per-cell policy can act on, and the prevailing protocol does not supply them. Benchmarks apply one missingness pattern at a time, and under a mask that is homogeneous in fault type and gap length every cell receives the same diagnosis, so a router reduces exactly to choosing one operator for the record. We verify this on six standard patterns and construct mixed masks in which transient loss, single-sensor outages and station blackouts occur together, as they do in a real record. A second correction proved necessary for the same reason. Evaluation segments drawn as contiguous blocks of complete cases are not contiguous in time, which silently inflates the real duration of a nominal gap, and a study that thresholds on duration cannot rely on them. Both are stated in Section 3.7 and Section 3.3.

We organize the contributions by the perspective from which each is evaluated.

(1). We place a single fixed operator, a hard router and a per-cell combiner inside one family of policies over the simplex of the operator pool, which allows the granularity question to be asked as a single question rather than as a comparison of unrelated methods. The family is restricted to policies whose entire input is the observation mask and the calendar, for the reason just given.

(2). We show that the useful mechanism is hedging rather than finer selection. A per-cell selector trained to pick one operator, with MAWC’s features and network size, is worse than MAWC at every held-out site, and so is the argmax of MAWC’s own weights. A convex average is also the only kind of policy that can fall below the regime oracle, which is a bound on every hard router using the same regimes, however good its diagnosis. We separate the contribution of the mask from that of averaging with interior-valued references that average without reading the mask, or read it only through the router’s regimes.

(3). We introduce two bounds that separate a deficient rule from a deficient action space, and two protocol corrections, without which, neither policy can be measured. Masks must mix fault types, for the reason given above. Evaluation segments must be contiguous in time: under the conventional construction from complete cases, on average, 3.6% to 33.9% of masked cells at Alice Springs, Yulara and Maine, and up to 58% in a single realization, sat in gaps that were longer in real time than their nominal length. Both corrections apply to any study that evaluates a rule over imputation operators rather than an operator alone.

(4). In experiments, both policies improve on the operator a deployer could have committed to in advance at all four held-out sites. The router reaches within 4.5% to 6.8% of its own ceiling at three of the four, which locates the remaining headroom in the action space rather than in the rule, and its threshold transfers across a fifteen-fold range of cadence, whereas a dispatch table fitted on the development sites does not. MAWC (reported as an ensemble of ten independently trained combiners, because a single one varies with its training seed by as much as the margins at stake) improves on the router at all four sites, significantly at three, and the comparison survives retrained deep members, doubled replication, changed fault composition and a higher missing rate.

## 2. Related Work

### 2.1. Imputation for Solar and PV Monitoring

Repair of PV monitoring records is treated as a processing stage in its own right rather than as preprocessing, because the choice made there propagates into performance indicators and degradation estimates [1,2]. Demirhan and Renwick [3] compared 36 univariate methods for horizontal irradiance and found the ranking to depend on both cadence and gap length, an early indication that a single recommendation does not hold everywhere. de-Paz-Centeno et al. [4] studied PV production under resource constraints, where the imputer’s own cost is a deployment criterion. What the literature establishes is that no operator dominates. The authors’ preliminary comparison mentioned in the Introduction sharpened that finding on three of the sites used here, attributing repair quality to the evidence that a fault leaves intact rather than to model capacity, and it stopped deliberately at measurement, selecting no operator and proposing none. Recent studies have also combined imputation with downstream PV forecasting [5] and compared machine learning gap filling for solar generation databases [6]. What none of this addresses is how a pipeline should choose among operators at run time, which is the question here.

### 2.2. Selecting or Combining Estimators

Choosing among predictors from features of the instance is old ground, and combining rather than selecting is older still. Forecast combination has been known to beat its components since Bates and Granger [7]; stacked generalization [8] with its regression form [9] learns a second-stage model over base-learner outputs; mixtures of experts [10] learn a gate that weights specialists by input; and algorithm selection [11] frames the choice as a mapping from problem features to algorithms. MAWC is an instance of that family and we claim no novelty for its form.

What distinguishes the present setting is the information that the gate is allowed to read. In a standard stacking or gating problem, the features describe the input, and the input is available. Here the quantity to be repaired is by definition absent, so the feature map is restricted to the mask and the calendar, and the paper’s question becomes how much of the achievable gain survives that restriction. To our knowledge, this constraint has not been imposed in work on estimator selection for imputation. The weighting mechanism is therefore not what this paper contributes. Its contributions are the deployability constraint, a feature map built from the mask alone, and an evaluation protocol that can measure a rule over operators: vertex-valued oracles that separate a deficient rule from a deficient action space, interior-valued references that separate the mask’s contribution from that of averaging, and the two corrections of Section 3.3 and Section 3.7.

### 2.3. Deep Imputation and What It Leaves Open

Deep sequence imputers learn temporal and cross-variable structure jointly. GRU-D and BRITS [12,13] use recurrent dynamics with learnable decay; SAITS uses self-attention with a joint reconstruction and imputation objective [14]; ImputeFormer adds a low-rank inductive bias to the transformer [15]; graph neural imputers such as GRIN propagate information between related sensors [16]; adversarial [17] and diffusion-based [18] imputers instead model the predictive distribution; and conditional diffusion has recently been specialized to spatiotemporal solar irradiance [19]. A recent survey organizes these methods by architecture and by how they treat imputation uncertainty [20]. Benchmarks such as TSI-Bench [21] have standardized their evaluation across many datasets, but have done so under a masking protocol that applies one missingness pattern at a time. Any of these methods can enter the pool as an operator; the policy studied here is agnostic to what the operators are.

That protocol is adequate for ranking operators and inadequate for evaluating a policy over them, for a reason that is arithmetic rather than empirical: if every masked cell in a record belongs to the same fault type and has the same gap length, a regime-based router has nothing to decide and reduces exactly to choosing one operator for the whole record, and a per-cell policy can vary its choice only through features unrelated to the fault, such as the time of day. Section 3.7 makes the point quantitatively and constructs the mixed masks a policy has to be measured on.

## 3. Materials and Methods

### 3.1. Problem Formulation

Let $Y\in R^{n\times d}$ be a monitoring record of *n* time steps and *d* synchronized channels sampled at a constant interval, and let $M\in {\left\{0,1\right\}}^{n\times d}$ be the observation mask, with $m_{t,j}=1$ when $y_{t,j}$ is missing. Write $M=\{\left(t,j\right):m_{t,j}=1\}$ for the set of missing cells and $Y_{\mathrm{obs}}$ for the observed part of the record. A repair operator can be expressed as

(1) $$g:\left(Y_{\mathrm{obs}},\;M\right)\;⟼\;\hat{Y},\;{\hat{y}}_{t,j}=y_{t,j}\;\;\mathrm{for}\;\left(t,j\right)\notin M.$$

**Figure 1:**
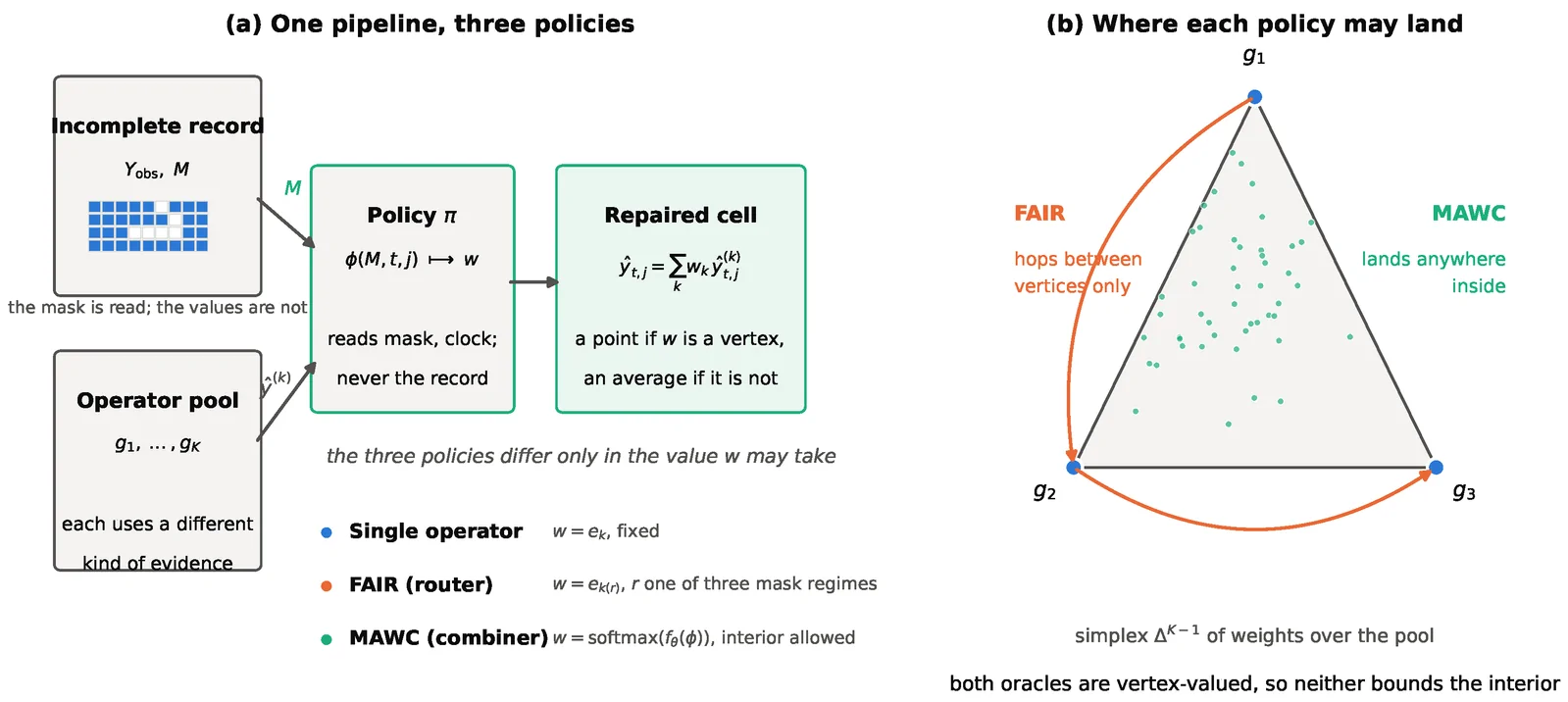
The family of policies studied. (**a**) All three read the same two inputs and produce a repaired cell the same way, and what distinguishes them is only the value the weight vector *w* in Equation (2) is permitted to take. The mask enters the policy and the record never does, which is what makes every member of the family deployable. (**b**) The same distinction as geometry. A single fixed operator is one vertex of the simplex, the router moves between vertices, according to which of three regimes the mask places a cell in, and the combiner may land anywhere inside. Both oracles defined below are vertex-valued, so they bound the first two classes and not the third.

Two properties of Equation (1) organize this paper, and both concern the mask rather than the values. The classical missing-data framework distinguishes mechanisms by how the probability of being missing depends on the data [22,23]. The distinction that matters operationally here is a different one. First, the mask *M* is an input: a monitoring system knows at repair time which cells are missing and nothing about their values, so any decision that depends only on *M* is available in deployment, while any decision that depends on *Y* at the masked cells is not. Second, different operators consume different subsets of $Y_{\mathrm{obs}}$, namely the same channel near *t*, the other channels at *t*, or the same channel at t−kP for the diurnal period, *P*. It follows that the operator which succeeds at a given cell is determined by which of those three kinds of evidence the fault has left intact. Given a pool of *K* operators, $g_{1},\ldots ,g_{K}$, a selection policy, π, assigns to every missing cell a distribution over the pool,

(2) $$\pi :\;\phi \left(M,t,j\right)\;⟼\;w\in \Delta^{K-1},\;{\hat{y}}_{t,j}^{\pi }=\sum_{k=1}^{K}w_{k}\;{\hat{y}}_{t,j}^{\left(k\right)},$$

where ϕ(M,t,j) is a feature map that reads the mask and the calendar but never the record. Equation (2) covers the whole spectrum this paper studies. A single fixed operator is the constant policy, $w=e_{k}$. A hard router is a policy whose output is always a vertex of the simplex and depends on *M* only through a small number of discrete regimes. A combiner is a policy whose output is anywhere in the simplex. Figure 1 shows the family and the geometry that separates its members.

Two quantities bound what any policy that outputs a vertex of the simplex can achieve on a given record, and we use both throughout. Neither is a policy: both require the withheld values, so they are ceilings rather than competitors. The per-cell oracle selects, for each missing cell independently and with knowledge of the truth, the pool member with the smallest error,

(3) $${\mathrm{RMSE}}_{\mathrm{cell}}={\left(\frac{1}{\left|M\right|}\sum_{(t,j)\in M}\min_{k}\;{\left({\hat{y}}_{t,j}^{\left(k\right)}-y_{t,j}\right)}^{2}\right)}^{1/2}.$$

The regime oracle is the corresponding quantity when the policy is constrained to be constant within each of the *R* regimes a hard router distinguishes,

(4) $${\mathrm{RMSE}}_{\mathrm{regime}}={\left(\frac{1}{\left|M\right|}\sum_{r=1}^{R}\min_{k}\sum_{\left(t,j\right)\in M_{r}}{\left({\hat{y}}_{t,j}^{\left(k\right)}-y_{t,j}\right)}^{2}\right)}^{1/2},$$

where $M_{r}$ is the set of missing cells assigned to regime *r*. The regime oracle is the ceiling of every hard router that uses the same regimes, including a router whose decision rule is perfect. The per-cell oracle is the ceiling of hard per-cell selection at any granularity.

The restriction to vertices is what makes the two quantities informative, rather than merely small. A policy in the interior of the simplex is bounded by neither, because a convex average of several estimators can lie closer to the truth than any one of them, so where a combiner falls below a bound, its gain is of a kind that no selection rule can reach, however finely it selects. Section 4.1 reports on where this happens.

### 3.2. Data

We use six photovoltaic monitoring records that differ in climate, sampling cadence and instrument set (Table 1). Two are used for development and four are held out entirely; the split is fixed in Section 3.8 and never revisited.

The two development stations are from the Desert Knowledge Australia Solar Centre public archive [24]. The four held-out records are systems 1239, 1276, 1278 and 1289 of the NREL PVDAQ collection, distributed through the Open Energy Data Initiative [25]. The two Las Vegas systems are distinct installations at the same location, which lets a site-to-site difference within one climate be distinguished from a difference between climates. Each record provides PV power together with synchronized environmental channels. Wind direction, where present, enters as its sine and cosine rather than in degrees, because the raw variable is circular and imputing it as a scalar would treat $1^{\circ }$ and $359^{\circ }$ as distant. Temperatures recorded in Fahrenheit or Kelvin and wind speeds in miles per hour are converted to SI, which leaves the standardized metric unchanged but keeps the physical clipping bounds meaningful. All series are used at their native cadence, and the diurnal period *P* is read from the modal sampling interval rather than assumed.

A seventh record, a subtropical rooftop installation in Hong Kong, was prepared and then excluded: its PV power channel is missing at 19.9% of timestamps, scattered throughout, so its longest stretch in which every channel is present without interruption is just under seven days, far below the minimum this protocol requires (Section 3.3). We report it separately rather than silently dropping it, and note that excluding it is the conservative choice, since it is the site at which the router’s margin was largest.

### 3.3. Evaluation Segments Must Be Contiguous in Time

A protocol detail that is usually invisible becomes load-bearing here, and we state it because it changed our own results. Imputation benchmarks commonly draw the evaluation window as a contiguous block of complete cases: rows in which every channel is present are collected, and a stretch of that collection is taken as the segment. Where rows were dropped for incompleteness, such a stretch is contiguous in row order but not in time. A block of *ℓ* consecutive positions then spans more than *ℓ* sampling intervals of real time, and by an amount that varies with how much of the record was dropped.

For a benchmark in which gap length is only a label, the distortion is tolerable. For this paper, it is not, because the router thresholds on gap duration and converts that threshold to physical hours, precisely so that it transfers between cadences (as shown in Equation (6)), and because the persistence ensemble reads the value at t−kP, which is the same clock time on an earlier day only if the intervening rows are all present. We reproduced the conventional selection with the same window and mask draws as our protocol. Averaged over 20 realizations, 3.6% of masked cells at Alice Springs, 33.9% at Yulara and 21.9% at Maine sat in gaps whose real duration exceeded their nominal one, and in a single realization, the share reached 58%. Most inflated gaps are only slightly longer than their label, but the tail is long: at Yulara, one nominal gap spanned 452 times its nominal duration (Appendix A, Table A1).

We therefore restrict each site to its longest stretch of usable rows that is also contiguous in time, and require that stretch to cover at least 20 days. The segment is drawn at least 20P positions into that stretch, so the 20-day lookback that the persistence ensemble needs lies inside it by construction. Every site in Table 1 then contains zero index discontinuities, verified rather than assumed. The cost is segment length: Yulara retains 15.7 days and Golden 13.9 days, against 69.4 and 177.7 days at the two largest sites, and Hong Kong retains too little to be used at all. Short segments limit what those two sites can show. At Yulara, the evaluation window fills the whole stretch after the 20-day lookback, so all realizations share one window and differ only in their masks, and each Golden window spans two weeks of a single season, so neither site says anything about how the policies behave across a year. We treat both as limitations (Section 5.5).

### 3.4. Operator Pool

The pool spans the three kinds of evidence identified in Section 3.1: the local signal, the contemporaneous channels and the diurnal repetition. Every member sees only the observed cells of the evaluation window and the record preceding it; no member reads a masked value, and no member is tuned on a held-out site or to favor any policy studied here. Standardization uses each channel’s mean and standard deviation over the complete rows of the site; this is a fixed scale rather than a fitted parameter.

- Linear interpolation (time-local): Each channel is interpolated linearly across its own gap; a gap touching the window edge takes the nearest observed value, and consumes the local signal only.
- Persistence ensemble (periodic): The values at the same clock time on each of the previous 20 days form an ensemble whose median is the estimate; a member that is itself missing or masked is replaced by the mean of the available members. It consumes the diurnal repetition only, and reaches back 20 days by construction, so it remains defined when every channel is missing at *t*. Seasonal-naive predictors are strong baselines because they encode a known period rather than learn it [26].
- MICE (cross-variable): Multiple imputation by chained equations [27] in the scikit-learn implementation (version 1.9.0) [28] (IterativeImputer, Bayesian ridge regression per variable, mean initialization, five round-robin iterations, posterior mean), fitted on the standardized evaluation window with the masked cells marked missing. Each variable is regressed on all others, so any pattern of missing channels is handled by the same model. It consumes the contemporaneous channels.
- Residual conditional Gaussian (periodic + cross-variable): A joint Gaussian on the standardized day-lag residual $y_{t}-y_{t-P}$, whose mean and covariance are estimated from the complete rows outside the evaluation window. A missing cell is imputed by the Gaussian conditional on the channels whose residual is observed at *t*, that is, whose value at both *t* and t−P is observed, and the conditional estimate is added to the most recent observed value at the same clock time. The conditional covariance is symmetrized and given a diagonal jitter of $10^{-8}$; the estimate is the median of 100 conditional draws.
- Conditional Gaussian (cross-variable): The same construction on standardized levels rather than residuals, conditioned on the channels observed at the same timestamp. Low-rank completion, in its iterative-SVD [29] and convex-relaxation [30] forms, is the other classical member of this family and is not carried; it was the weakest operator on these records in the authors’ preliminary comparison.
- SAITS (deep, attention): Self-attention imputation transformer [14] in PyPOTS 1.5 [31]—two layers, $d_{\mathrm{model}}=64$, four heads, $d_{k}=d_{v}=16$, feed-forward width 64, 20 epochs, and batch size 32. The evaluation window is cut into non-overlapping sequences of one diurnal period, and the model is trained self-supervised on those masked sequences, separately for each site and realization, with no held-out tuning.

Two considerations fixed the membership. The pool must contain at least one operator consuming each kind of evidence, so that no fault type is left without an operator that can use what survives it, and on the development sites alone, every member is the closest estimate at a non-trivial share of cells in at least one regime (Appendix B, Table A2). SAITS is included because it is the strongest or near-strongest single operator on the development sites, and excluding it would flatter every policy in this paper. Other deep imputers, such as BRITS and TimesNet [13,32], and the graph and diffusion models of Section 2.3, were not carried. Adding members can only raise the per-cell oracle and give a combiner more to hedge across, so the pool fixes the scale of every result here without biasing the comparison between policies, which see the same pool.

### 3.5. Policy 1: A One-Parameter Fault-Aware Router

The first policy diagnoses each missing cell from the mask alone and dispatches it to one operator. For channel *j*, let $\rho_{t,j}$ be the length of the contiguous missing run containing (t,j), and let $o_{t}=\left[\exists j^{\prime }:m_{t,j^{\prime }}=0\right]$ indicate that at least one channel is observed at time *t*. With a threshold, *L*, in the steps,

(5) $$r\left(t,j\right)=\left\{\begin{array}{cc} 0 & \rho_{t,j}\leq L\;(\mathrm{transient}\;\mathrm{loss})\\ 1 & \rho_{t,j}>L\;\mathrm{and}\;o_{t}\;(\mathrm{single}\text{-}\mathrm{sensor}\;\mathrm{fault})\\ 2 & \rho_{t,j}>L\;\mathrm{and}\;\neg o_{t}\;(\mathrm{station}\;\mathrm{blackout}) \end{array}\right.$$

and the router returns linear interpolation for regime 0, MICE for regime 1 and the persistence ensemble for regime 2. We refer to this policy as FAIR, for fault-aware imputation router; the name is unrelated to the FAIR principles for research data.

The dispatch table is the assignment of operator families to fault types found in the authors’ preliminary comparison, and that comparison included Maine, one of our held-out sites. The table therefore carries information from a held-out site, although no parameter of the router was fitted there. To remove this dependence, we also report a development-only router with the same diagnosis and threshold, whose table assigns to each regime the pool member with the lowest error on the development sites. It sends transient loss to linear interpolation and single-sensor faults to MICE, as FAIR does, and blackouts to SAITS rather than to the persistence ensemble. MAWC reads no dispatch table at all, so none of its results depend on this choice.

Two aspects of Equation (5) are deliberate. First, the distinction between regimes 1 and 2 is not a matter of gap length but of whether any contemporaneous evidence survives, which is the property that determines whether a cross-variable operator can function at all. Second, the threshold is specified as a physical duration *T* in hours and converted per site,

(6) $$L=\mathrm{round}\;\left(T\;P/24\right),$$

rather than as a fixed number of steps. A threshold fixed in steps is a different fraction of a day at every cadence and so cannot transfer between sites, whereas Equation (6) makes the same rule meaningful at 5 min and at 15 min. *T* is the policy’s only free parameter, and Section 3.8 states how and where it is calibrated.

### 3.6. Policy 2: MAWC—Mask-Aware Weighted Combination

The second policy, which we call MAWC, replaces the hard three-way choice with a weight over the whole pool, predicted per cell from the same kind of information. A network, $f_{\theta }$, maps the feature vector to unnormalized scores, and the weights are their softmax,

(7) $$w\left(t,j\right)=\mathrm{softmax}\left(f_{\theta }\left(\phi \left(M,t,j\right)\right)\right),\;{\hat{y}}_{t,j}=\sum_{k=1}^{K}w_{k}\left(t,j\right)\;{\hat{y}}_{t,j}^{\left(k\right)}.$$

Three properties of Equation (7) are chosen rather than inherited. The softmax constrains the weights to the simplex, which keeps the output a convex combination of estimators already calibrated in physical units, so the prediction cannot leave the range the pool spans and no separate scale has to be learned. The pool enters only through ${\hat{y}}^{\left(k\right)}$ and never through ϕ, so $f_{\theta }$ cannot recover a value it is meant to predict and the deployability argument of Section 3.1 survives training. $f_{\theta }$ is deliberately small, a single hidden layer of 16 units with tanh activation giving 294 parameters at K=6, because the quantity being predicted is how far each operator can be trusted given a handful of mask statistics. That is a low-dimensional function, and a larger network would risk fitting the development sites rather than the relationship. Section 4.5 reports the learning curve this choice predicts.

Training minimizes the squared error of the combined prediction over the missing cells of the development sites, where the withheld values are known because the masks are synthetic (Section 3.7). Features are standardized with the mean and standard deviation of the development cells. The network is initialized with the PyTorch (version 2.9.0) defaults and optimized by Adam with learning rate 0.02, mini-batches of 8192 cells and 200 epochs over the 299,093 development cells, with no weight decay, no validation split and no early stopping; the epoch count is fixed in advance and the training error has flattened well before it. No held-out value enters the loss or any choice about training.

A single network of this size is not stable across training seeds: on the held-out sites, its error varies between seeds by up to 0.034 in RMSE, as large as some of the margins this paper interprets (Appendix C). We therefore report MAWC as an ensemble of ten networks trained under different seeds on the same development cells, whose predicted weights are averaged cell by cell. An average of convex weights is itself convex, so the ensemble remains a policy of the form Equation (2), and reads nothing beyond the mask and the calendar. Two ensembles built from disjoint sets of seeds agree to within 0.005 at every held-out site (as shown in Table A3). The features are listed in Table 2. All features derive from the mask and the calendar and none reads a value, so Equation (7) is as deployable as Equation (5).

The feature map is fixed before any held-out site is loaded. Its design is deliberately conservative: every entry is either a quantity the router already uses, expressed continuously instead of thresholded, or a quantity whose relevance follows from the evidence argument of Section 3.1.

### 3.7. Missingness Patterns

Evaluating a per-cell policy requires masks in which a single record contains more than one fault type, and this requirement is not satisfied by the pattern-at-a-time protocol usual in imputation benchmarks. Under a mask that is homogeneous in fault type and gap length, every cell receives the same diagnosis, so a regime-based router reduces exactly to choosing one operator for the whole record and its per-cell behavior is never exercised. We verified this on six standard patterns (5% random dropout, single-sensor outages of 3 h, 12 h, 1 day and 3 days, and a one-day all-channel blackout), each of which has a single fault type and a single gap length: each yields a regime share of 1.000 for one regime and 0.000 for the other two, and FAIR’s error equals that of the operator its table assigns to that regime to five decimals. A per-cell policy such as MAWC is not reduced exactly, since it can still vary its weights with time of day, channel and position within a gap, but under such a mask, it is never asked to distinguish one fault type from another, which is the decision this paper is about.

We therefore construct a mixed mask in which all three fault types are present simultaneously, as they are in a real record:

- Transient loss: each cell removed independently with probability α;
- Single-sensor faults: contiguous blocks in one randomly chosen channel, at three durations (3 h, 12 h, 1 day), while all other channels keep reporting;
- Station blackouts: contiguous blocks of one day in which every channel is removed.

The number of blocks of each kind is not fixed in absolute terms but solved from the site’s own period and channel count so that each regime receives approximately the same share α of all cells at every site,

(8) $$n_{\mathrm{sensor}}\left(\ell \right)=\left\lceil \frac{\alpha \;n\;d}{3\;\ell }\right\rfloor ,\;n_{\mathrm{blackout}}=\left\lceil \frac{\alpha \;n\;d}{P\;d}\right\rfloor ,$$

for a single-sensor block of length *ℓ*. Without Equation (8), a fixed block count would give a 15 min site and a 5 min site different regime compositions, and a site with seven channels a different composition from one with five, so the sites would not be comparable. We set α=0.03, which yields a total missing fraction near 8.5%.

The realized shares are not equal thirds and we report them rather than the design target. At the three 15 min sites they average 0.44, 0.23 and 0.34 for the transient, single-sensor and blackout regimes, and no regime’s share varies by more than 0.03 between those sites, which is the stability comparability requires. The departure from equal thirds follows from Equation (5) rather than from Equation (8): a single-sensor block overlapping a blackout is counted as a blackout cell, and a transient cell adjacent to a block joins that block’s run, so cells migrate out of the single-sensor regime in both directions. That overlap is permitted, and resolving it in favor of regime 2 is the correct reading, since when no channel is observed, no cross-variable evidence exists, whatever caused the cell to be missing. The exception is the 1 min site, where the blackout share reaches 0.55 because a single one-day blackout spans 1440 steps and already exceeds the target, and Equation (8) cannot place a fraction of a block. Section 4.6 shows that this is not a cosmetic difference.

Regarding how the synthetic masks relate to the records’ own gaps, synthetic masks are necessary because the policies must be scored against values that were withheld rather than lost, but they are not a model of any particular plant. The records’ own missingness, diagnosed by the same rule Equation (5) on a regular time grid (Appendix A, Table A1), affects between 0.7% and 7.1% of cells and is far more uneven in composition than our design. Station-wide outages dominate at Golden and at Las Vegas (a), single-sensor faults at Hong Kong and Yulara, and transient loss at Las Vegas (b). We therefore set α so that the total missing fraction exceeds every natural rate and each regime receives enough cells to be measured, and we treat composition and rate as variables rather than as fixed properties. Section 4.7 repeats the comparison at α=0.01 and α=0.06 and with blackout-dominated and sensor-fault-dominated compositions. What synthetic masks cannot reproduce is the dependence of real faults on the values themselves, such as inverter trips at high power or icing in cold weather, together with their seasonality and correlation across sites. These remain outside the scope of this study (Section 5.5).

### 3.8. Evaluation Protocol

Alice Springs and Yulara are development sites. Maine, the two Las Vegas systems and Golden are held out: each is loaded, predicted once and reported. No held-out site contributes to the router’s threshold, MAWC’s weights, the feature map, the mask design or any stopping decision. Two inherited choices are the exception, because they come from the authors’ preliminary comparison, which included Maine: FAIR’s dispatch table and the omission of low-rank completion from the pool. The development-only router of Section 3.5 replaces the first, and Appendix B re-derives the pool’s composition from the development sites alone.

The router’s threshold, *T*, is selected on a grid over {0.5,1,2,4,6,8,10,14,20} hours, on the development sites only, by mean error. The selected value is T=8 h, which sits at the center of a flat optimum: 6, 8 and 10 h give the same development error to four decimals, and the error rises on both sides of that plateau. The value is then frozen and applied unchanged at all three cadences via Equation (6), giving L=96 steps at 5 min, 32 at 15 min and 480 at 1 min.

Comparing a policy against the best operator at the held-out site would credit it with information no deployer has, since that operator is identifiable only after the withheld values are revealed. We therefore report the pre-committed operator: the pool member with the lowest mean error on the development sites under the same mask distribution, chosen before any held-out site is touched. The hindsight-best operator at each held-out site is also reported, but as a reference rather than as the comparison the paper rests on. The pre-committed choice is itself fragile. On the development sites linear interpolation and SAITS are close, and because SAITS is trained stochastically, a rebuilt pool can reverse their order, as happens in several configurations of Section 4.7. We report whichever operator the development sites select in each configuration, and read the comparison with it as a comparison with a reasonable deployer rather than with a fixed opponent.

All errors are root mean squared errors over masked cells in per-variable *z*-units, each channel divided by its own standard deviation computed from the complete rows of that site. Standardization is necessary because the channels differ by orders of magnitude in physical units, so an unstandardized average would be dominated by whichever channel has the largest scale and would not be comparable across sites with different instrument sets. Because PV power is usually the variable a downstream consumer cares about most, Appendix D also reports MAE, RMSE on the PV channel alone and on daytime PV cells, and the error of the PV energy recovered over the missing cells, a downstream quantity in physical units. We score point accuracy only. Three pool members return an ensemble and could be scored by a strictly proper rule such as the continuous ranked probability score [33], but MAWC returns a single value by construction, so a probabilistic comparison would not cover the policies this paper is about.

Each site is evaluated over *S* independent mask realizations. Every policy and every pool member sees byte-identical masks, drawn from the same generator seeded per realization, so all comparisons are paired at the level of the realization. Differences are reported with a 95% percentile interval from 20,000 paired bootstrap resamples over realizations, rather than as point estimates alone. The pool’s outputs are computed once and cached, so every policy, reference and bound in the tables of Section 4 reads the same pool outputs on the same masks, which is what makes their columns mutually paired. The deep pool member is trained stochastically; Section 4.7 retrains it under two further seeds and rebuilds the pool.

## 4. Results

All figures are the mean of per-realization RMSEs over 20 mask realizations, the same quantity the paired tests are computed on. We note the convention because the alternative, pooling every realization’s cells into one RMSE, differs by 0.1% at the most stable site and by 7% at the most variable one, which is enough to change how a table reads. MAWC denotes the ten-network ensemble throughout; Appendix C reports single networks.

### 4.1. The Evidence Ladder

**Figure 2:**
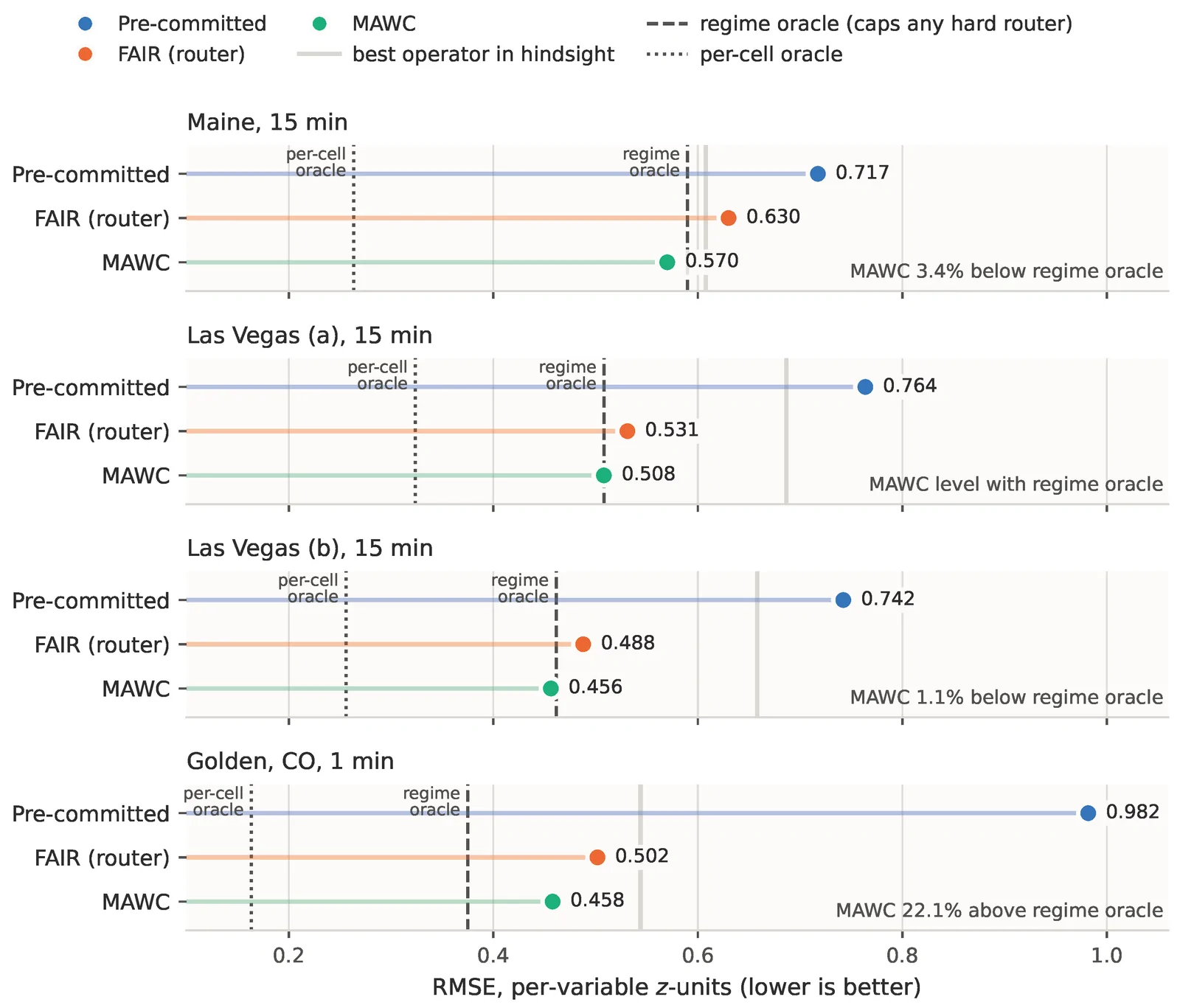
The evidence ladder at each held-out site. Filled markers are the three policies a deployer can actually run, since each reads only the observation mask. The vertical rules require the withheld values and therefore serve as bounds rather than as competitors: the dashed rule is the regime oracle, which caps every hard router using the router’s three regimes, however good its diagnosis, and the dotted rule is the per-cell oracle. Each rule is labeled in every panel. All values are means over 20 mask realizations in per-variable *z*-units.

Table 3 and Figure 2 place both policies between what a deployer could have chosen in advance and the two bounds that require the withheld values.

Reading the deployable columns first, the pre-committed operator, linear interpolation, is the weakest choice at every site. The router improves on it everywhere, by 12% at Maine and by 49% at Golden, and MAWC improves on the router at all four sites in the mean, by 4.3% to 9.5%.

The bound columns make those differences interpretable. At Maine and the two Las Vegas systems, the router sits 4.5% to 6.8% above the regime oracle, so at those sites the diagnosis has little left to give and further gains must come from the action space rather than from the rule. At Golden, it sits 33.8% above; Section 4.6 shows that most of that distance reflects how loose the oracle becomes at that site. MAWC falls below the regime oracle at Maine (−3.4%, significant in the paired comparison) and at Las Vegas (b) (−1.1%), and matches it at Las Vegas (a) (Section 4.3). Crossing that bound is possible only because a convex average is not a selection, and it is the clearest evidence that the gain is not of a kind that a hard router could reach, however good its diagnosis. Both policies remain far from the per-cell oracle, by factors between 1.6 and 3.1, so most of the theoretical headroom is not reachable by a decision that reads only the mask.

### 4.2. Paired Comparisons

Point estimates alone would overstate the case, so Table 4 reports each comparison paired at the level of the mask realization.

MAWC is significantly better than the pre-committed operator, the hindsight-best operator and the development-only router at all four sites. Against FAIR, it is significantly better at Maine and at the two Las Vegas systems, winning 20 of 20 realizations at Maine but 14 and 16 of 20 at Las Vegas, where the margin is real but small. At Golden, the comparison with FAIR does not resolve: MAWC wins 11 of 20 realizations and the interval spans zero.

### 4.3. What the Mask Adds Beyond Averaging

Both oracles are vertex-valued, so falling below them shows that interior weights help but not that reading the mask does: a combiner could gain simply by averaging. The references of Table 5 separate the two. Uniform weights, a single set of convex weights fitted on the development sites, and unconstrained linear stacking all ignore the mask, and MAWC is significantly better than each of them at every held-out site. Averaging without the mask is therefore not what produces the gain.

Per-regime convex weights are the harder reference, since they hedge within each of FAIR’s regimes and read the mask only through the diagnosis. They already improve on FAIR by 8.4% in Maine, where they reach below the regime oracle as well, and by 0.9% and 1.7% in the Las Vegas systems. MAWC is significantly better than them at all four sites, by 0.007 in Maine and by 0.018 to 0.047 elsewhere, so the finer mask features that MAWC reads and the diagnosis discards contribute beyond hedging within the router’s regimes.

### 4.4. Where the Gain Comes from

The natural reading of a per-cell policy is that it wins by deciding more finely. Table 6 tests that reading with two hard selectors built on MAWC’s feature map. The argmax of MAWC’s own weights is one, but it is read off networks trained to blend, which handicaps it as a selector. The other is an ensemble of selectors trained by cross-entropy to pick the operator with the smallest error in each development cell, which is the natural way to train for selection. Both lose at every site: the argmax by 14.5% to 26.5%, the trained selector by 14.6% to 34.4%. A per-cell decision rule with the same information does not recover MAWC’s accuracy, however it is trained, so the gain is hedging rather than granularity.

The feature groups in Table 6b rank consistently with the evidence argument of Section 3.1. Gap geometry, the length of the containing gap and the position within it, carries most of the decision, and removing it degrades MAWC significantly at all four sites. Channel identity follows. The periodic-availability features and the interaction of gap length with the absence of contemporaneous evidence each change the mean by less than 1% and are significant at one site only; they are close to redundant with the rest of the feature map in the present pool. We keep them because the feature map was fixed before any held-out site was loaded, and removing features on held-out evidence would break that commitment.

### 4.5. Training Size

Raising the number of realizations from 5 to 20 quadrupled MAWC’s training set while leaving the router’s single parameter untouched, so part of the margin might have been extra training signal rather than a better policy. Table 7 repeats the training at seven sizes, under five seeds each.

Held-out error falls from the smallest training set to roughly 15,000 development cells and changes little thereafter. Beyond that point the differences between sizes are of the same order as the differences between seeds, so the table supports a statement about how much data the policy needs, not a claim that accuracy is exactly flat. The comparison in Table 3 does not depend on the full size of the training set.

### 4.6. Regimes and the One-Minute Site

Golden is the only record in the study at 1 min cadence, fifteen times finer than the sites the rules were calibrated on. Table 8 breaks the error down by regime.

The threshold transfers. Expressed as a physical duration and converted per site by Equation (6), it separates the regimes as designed at every cadence, and at Golden, FAIR’s assignment is the best single operator on average in the transient and single-sensor regimes and within 1% of the best in the blackout regime. The router’s distance from the regime oracle there has a different source. At 1 min cadence, a one-day blackout spans 1440 steps, so the blackout cells of a realization form a single whole day and make up 55% of the masked cells, against roughly a third elsewhere. Which operator repairs a given day best depends on that day: across the 20 realizations, the best operator for Golden’s blackout cells is the persistence ensemble 9 times, the residual conditional Gaussian 7 times, linear interpolation 3 times and SAITS 1 time. The regime oracle chooses in hindsight for each realization, and no rule reading only the mask can, so at this site the oracle is loose rather than the router deficient.

A table fitted where the rule was calibrated need not transfer, however. The development-only router sends blackouts to SAITS, the best choice on the development sites, and at Golden that choice costs 14% against FAIR. MAWC does not depend on a table. It improves on FAIR in the blackout regime, where hedging across operators whose ranking changes from day to day pays, and is much worse than FAIR on single-sensor faults, where MICE is far better than every other member and spreading weight away from it costs accuracy. The two effects roughly cancel, which is why the comparison with FAIR does not resolve at Golden, while MAWC remains significantly better than every other deployable reference there.

### 4.7. Sensitivity to the Pool, the Replication and the Masks

Table 9 rebuilds the pool and retrains the ensemble under seven changed configurations. Retraining SAITS under two further seeds changes no conclusion, and neither does doubling the replication to 40 realizations. Changing the fault composition towards blackouts or towards single-sensor faults leaves the pattern intact: MAWC is significantly better than FAIR at Maine and at both Las Vegas systems, and does not resolve at Golden. Raising the missing rate to α=0.06 strengthens the result, and MAWC then lies significantly below the regime oracle at three sites. At α=0.01, the result is weaker. MAWC remains significantly better than FAIR at Maine, while at the other sites, the development sites supply less than half as many training cellsand far fewer blackout is not distinguishable from it at the two Las Vegas systems, and is significantly worse at Golden, by +0.099 with interval [+0.023,+0.178].

Three explanations are possible for the loss at Golden: fewer development training cells, a different balance of fault regimes, and Golden’s short single-season segment. Because the mask features are expressed in physical hours, a combiner trained at one missing rate can be applied at another, which separates the first two. Trained on the reference development set (α=0.03) and applied to the α=0.01 masks, MAWC is no longer significantly worse than FAIR at Golden (+0.035, interval [−0.024,+0.094]) and is again significantly better at Las Vegas (a). Subsampling the reference development set to the size of the α=0.01, one leaves this unchanged (+0.037), so the number of training cells is not the cause. What differs is the composition of the training set: the generator places at least one whole-day blackout in every record, so at α=0.01 half of the development cells are blackout cells, against 37% in the reference configuration, and the two policies are closer. Weights learned on that mixture transfer poorly. This accounts for roughly two thirds of the loss. The remainder cannot be attributed further. At α=0.01, two thirds of Golden’s masked cells belong to a single blackout day per realization within a two-week segment, and the present data do not separate the effect of that evaluation mask from the effect of the short segment. In the configurations where the development sites select SAITS rather than linear interpolation as the pre-committed operator, the router is no longer significantly better than that operator at Maine, where SAITS is strong; MAWC remains significantly better at every site in every configuration.

### 4.8. Further Analyses

Four further analyses address specific questions; their figures are in Appendix D and Appendix E.

*Supplementary metrics:* MAE orders the policies as RMSE does at every site (Table A4). On the PV channel alone, and on daytime PV cells, MAWC has the lowest error at three sites; at Las Vegas (b), FAIR is marginally lower on both. The error of the PV energy recovered over the missing cells, a downstream quantity in physical units, is significantly lower under MAWC than under FAIR at Golden (24% against 36%) and not significantly different at the other three sites. Its reduction relative to the pre-committed operator is large everywhere.

*What the mask-only restriction costs:* A combiner whose feature map also reads observed context through the pool’s own outputs, namely each member’s estimate, the pool’s spread and each member’s deviation from the pool median, is worse than mask-only MAWC at every site, significantly at three (Table A5a). It fits the development sites more closely and transfers worse. This does not show that no value-derived feature can help, but the natural ones do not, so the restriction costs nothing we could measure.

*Loss:* Training with a Huber loss instead of squared error changes held-out RMSE by less than 2% at every site, making it slightly worse at three and slightly better at Golden (Table A5a). The results do not hinge on the loss.

*Training on a held-out site:* A combiner trained on either Las Vegas system and applied to the other sites does at least as well as the development–site combiner everywhere except Maine, whereas one trained on Golden transfers poorly to the rest (Table A5b). Each site is repaired best by a combiner trained on its own masks, so what a combiner learns is partly specific to the site it was trained on. The development–site combiner of the main results is therefore a conservative choice. Where a site’s own history can be masked synthetically, fitting on it is worth doing.

## 5. Discussion

### 5.1. Why Hedging Beats Choosing, and When It Does Not

Neither the argmax of MAWC’s weights nor a selector trained for selection matches MAWC at any held-out site, so MAWC’s advantage is not that it decides more finely than the router but that it declines to decide. The mask supports a statement about how far each operator can be trusted, not about which one is right, and acting on a degree of trust is a different operation from choosing. It is also the only operation that can place a policy below the regime oracle, as it does at Maine: no selection rule, at any granularity, can imitate an average lying closer to the truth than each of its terms.

The single-sensor regime at Golden points to a natural extension. There, MICE is much better than every other member, and MAWC, which learned from the development sites how far to hedge, spreads weight that a hard choice would have concentrated. A policy that also learns when to commit would combine the router’s strength with the combiner’s.

### 5.2. What Transfers Between Sites

A quantity defined in physical units transfers, because the units mean the same thing at every site. The router’s threshold separated the regimes as designed at all three cadences. A choice fitted where the rule was written need not transfer, because its correctness is a property of the site. The development-only router’s blackout assignment is the clearest case: right on the development sites and 14% worse than the alternative at Golden. MAWC holds its dispatch as something to be inferred from mask features and is significantly better than the development-only router at all four held-out sites, but it inherits the development sites’ view of how far to hedge, which is what fails at Golden in the single-sensor regime.

### 5.3. Where the Advantage Is Not Established

Two results limit the claim and should be read alongside it. First, the improvement of MAWC over FAIR is statistically supported at three held-out sites, not four. At Golden, the mean difference favors MAWC (−0.044) but its interval, [−0.109,+0.019], includes zero, and doubling the replication to 40 realizations does not resolve it (−0.014). Wherever this paper says that MAWC improves on the router at all four sites, the statement concerns the mean; the statistically supported statement is three of four.

Second, the advantage depends on the missing rate. At α=0.01, MAWC remains significantly better than FAIR at Maine, is indistinguishable from it at the two Las Vegas systems, and is significantly worse at Golden (+0.099). The cross-rate analysis of Section 4.7 attributes most of that loss to the composition of the low-missingness training set, which is dominated by blackout cells, rather than to its size, and a combiner trained at the reference rate removes the significant loss. The part that remains may reflect Golden’s blackout-dominated evaluation masks or its short single-season segment, and the present data cannot distinguish the two. At low missingness, a fixed dispatch table is therefore a competitive choice, and a deployer who expects sparse faults should train the combiner on masks with a balanced regime composition rather than on masks drawn at the expected rate.

### 5.4. Scope Beyond Photovoltaic Monitoring

Nothing in the formulation of Section 3.1 is specific to photovoltaics. The policies read a mask and a calendar, the oracles and interior-valued references are defined for any pool, and the two protocol corrections apply to any study that evaluates a rule over imputation operators. What is specific is the evidence structure. Diurnal periodicity makes the persistence ensemble and the day-lag features meaningful, and a small number of co-located channels makes the distinction between single-sensor faults and blackouts sharp. In environmental and building sensing, which share both properties, we would expect the approach to carry over with the same pool. In domains without a dominant period, or with hundreds of channels, the regimes and the pool would have to be redefined, and whether hedging still beats choosing is an empirical question. The policy itself is agnostic to what the operators are, so a different pool changes the ceiling rather than the method.

### 5.5. Limitations

The masks are synthetic and missing completely at random within each fault type. They reproduce the three fault types but not the dependence of real faults on the values; their seasonality, or their correlation across channels and sites, and the records’ own gaps differ from our design in rate and composition (Table A1). The sensitivity analysis varies in rate and composition, and at the lowest missing rate MAWC is no longer better than FAIR outside Maine and is significantly worse at Golden (Section 5.3). The pool is fixed at six members, so the per-cell oracle over it is the ceiling of the whole exercise. The evaluation is of repair accuracy, with one downstream quantity; effects on degradation rates, performance ratios or forecasts are not measured. Four held-out sites span two climates and three cadences, the Yulara and Golden segments are short and cover a single season, and the 1 min cadence is represented by one site. The deep pool member is trained stochastically; we retrained it under three seeds in total, no more. A single combiner varies with its training seed, which is why we report an ensemble. Finally, two inherited design choices come from the authors’ unpublished preliminary comparison, which included one held-out site; we re-derived them from the development sites and report the router that results.

## 6. Conclusions

We asked how fine a repair decision has to be when the only information available at repair time is the observation mask, and answered it by placing two mask-only policies between what a deployer could have committed to in advance, two oracles that require the withheld values, and interior-valued references that average without reading the mask or read it only through a diagnosis. A one-parameter router, whose single threshold is a physical duration calibrated on two desert sites, improves on the pre-committed operator at all four held-out sites and reaches within 7% of the ceiling of its own action space at three of them. MAWC, an ensemble of small networks reading only mask and calendar features, improves on the router in the mean at all four sites, with significant improvements at three. The improvement is statistically supported at three of them, Maine and the two Las Vegas systems, and is not resolved at Golden. MAWC improves significantly on every interior-valued reference at all four sites, and falls below the regime oracle at two, in the mean at two sites, significantly at one, which no hard router using those regimes can do at any granularity.

The answer, however, is not that the decision must be finer. A per-cell selector with MAWC’s features, trained for selection, loses 15% to 34% against MAWC at every site. What the mask supports is a statement about how far each operator can be trusted, and acting on that degree of trust is a different operation from selecting the operator that looks best.

The advantage has a boundary. At a missing rate of α=0.01, MAWC remains significantly better than the router at Maine, is indistinguishable from it at the two Las Vegas systems, and is significantly worse at Golden. Most of that loss follows from the blackout-dominated composition of the low-missingness training set rather than from its size, and the rest cannot be separated from Golden’s short single-season segment. The case for MAWC is therefore strongest where the missingness is substantial.

Two protocol corrections were necessary before either policy could be measured, and both apply beyond this study. A mask homogeneous in fault type and gap length reduces a regime-based router to a single global choice and never asks a per-cell policy to distinguish faults, so a benchmark that applies one missingness pattern at a time can evaluate operators but not a policy over them. An evaluation segment assembled from complete cases is not contiguous in time, which inflated the real duration of up to 58% of masked cells in a single realization at the sites we measured, so any conclusion that depends on gap duration or on same-clock-time lookups needs segments that are constructed differently.

The scope of these conclusions, including the synthetic masks, the fixed pool and the number of sites, is set out in Section 5.5. For a monitoring pipeline, the practical reading is that the policy is cheap. It reads only the mask, each of its networks holds 294 parameters and trains in under a minute, and it can be fitted where withheld values can be simulated and deployed where they cannot.

## Figures and Tables

**Table 1:** Monitoring records; “dev.” marks a development site. Cadence spans a factor of fifteen and the channel count a factor of two, so a rule calibrated on the two desert stations has to survive both changes to be useful. The segment column is the evaluation window actually available at each site under the contiguity requirement of Section 3.3. Las Vegas (a) and (b) are PVDAQ systems 1276 and 1278, two installations at the same location.

Site	Role	Climate	Cadence	Chan.	Segment (d)
Alice Springs	Dev.	Desert	5 min	7	69.4
Yulara	Dev.	Desert	5 min	5	15.7
Maine	Held out	Cold continental	15 min	5	177.7
Las Vegas (a)	Held out	Hot desert	15 min	8	161.9
Las Vegas (b)	Held out	Hot desert	15 min	8	161.9
Golden, CO	Held out	Cold continental	1 min	4	13.9

**Table 2:** The feature map, ϕ(M,t,j). Durations are expressed in physical hours, and the run-length quantities are converted from steps using the site’s period, for the same reason that the router’s threshold is (Equation (6)).

Feature	Definition and Rationale
log(1+ρ/h)	Length of the containing gap in hours; the quantity the router thresholds
Position in run	Distance to the nearest edge of the gap, relative to half its length; interpolation is near-exact at a gap’s edges and worst at its center, a distinction the router cannot express
Edge distance (rel.)	The same distance relative to the full gap length
log(1+e/h)	Absolute distance to the nearest observed sample of this channel, in hours
Observed-channel fraction	Share of channels visible at *t*; the continuous form of the router’s $o_{t}$
Is PV power	Indicator for the power channel, whose behavior differs from the meteorological channels
sin,cos of hour	Time of day; night cells are nearly free for every operator
Day-lag observed	Whether $y_{t-P,j}$ was observed, a precondition for the persistence ensemble
Channel visibility, ±1 day	Share of this channel observed in a one-day window around *t*
Long gap × station dark	Interaction of gap length with the absence of contemporaneous evidence

**Table 3:** Held-out performance, RMSE in per-variable *z*-units, mean over 20 mask realizations with the standard deviation across realizations in parentheses. All columns are computed on byte-identical masks and pool outputs. MAWC is the ensemble of ten combiners (Section 3.6). The pre-committed operator (linear interp.) is the pool member with the lowest development-site error; the hindsight-best operator is identifiable only after the withheld values are revealed, and both oracles require the truth. Lower is better; the best deployable entry per site is in bold. Paired intervals are shown in Table 4. Las Vegas (a) and (b) are PVDAQ systems 1276 and 1278, two installations at the same location.

Site	Pre-Committed	FAIR	MAWC	Hindsight-Best	Regime Oracle	Per-Cell Oracle
	Deployable			Require the Withheld Values		
Maine	0.717 (0.05)	0.630 (0.03)	**0.570** (0.02)	0.608 (SAITS)	0.590	0.263
Las Vegas (a)	0.764 (0.11)	0.531 (0.16)	**0.508** (0.16)	0.687 (SAITS)	0.508	0.324
Las Vegas (b)	0.742 (0.11)	0.488 (0.15)	**0.456** (0.16)	0.658 (SAITS)	0.462	0.256
Golden, CO	0.982 (0.52)	0.502 (0.19)	**0.458** (0.12)	0.544 (resid. cond. Gauss.)	0.375	0.163

**Table 4:** Paired comparison of MAWC against each deployable reference and the hindsight-best operator, over the 20 mask realizations. Every policy sees byte-identical masks, so the comparison is paired at the realization level. Mean difference is MAWC minus the reference (negative favors MAWC); intervals are 95% percentile intervals from 20,000 paired bootstrap resamples over realizations. The development-only router uses a dispatch table fitted on the development sites (Section 3.5). Las Vegas (a) and (b) are PVDAQ systems 1276 and 1278, two installations at the same location.

Site	Comparison	Wins	Mean Diff.	95% CI
Maine	vs. FAIR	20/20	−0.060	[−0.070, −0.050]
	vs. dev.-only router	20/20	−0.061	[−0.074, −0.048]
	vs. hindsight-best	18/20	−0.038	[−0.051, −0.025]
	vs. pre-committed	20/20	−0.147	[−0.166, −0.127]
Las Vegas (a)	vs. FAIR	14/20	−0.023	[−0.040, −0.008]
	vs. dev.-only router	14/20	−0.038	[−0.058, −0.019]
	vs. hindsight-best	20/20	−0.179	[−0.211, −0.147]
	vs. pre-committed	20/20	−0.256	[−0.294, −0.217]
Las Vegas (b)	vs. FAIR	16/20	−0.032	[−0.050, −0.011]
	vs. dev.-only router	18/20	−0.045	[−0.062, −0.025]
	vs. hindsight-best	20/20	−0.202	[−0.234, −0.172]
	vs. pre-committed	20/20	−0.286	[−0.318, −0.252]
Golden, CO	vs. FAIR	11/20	−0.044 ^†^	[−0.109, +0.019]
	vs. dev.-only router	16/20	−0.115	[−0.165, −0.065]
	vs. hindsight-best	12/20	−0.086	[−0.167, −0.009]
	vs. pre-committed	19/20	−0.524	[−0.737, −0.328]

^†^ interval includes zero.

**Table 5:** Interior-valued references: Each is fitted on the development sites only and is deployable; the first three ignore the mask entirely, and per-regime convex weights read it only through FAIR’s three regimes. Entries are held-out RMSE with, in parentheses, MAWC minus the reference; ^†^ marks a paired 95% interval that includes zero. The per-regime row isolates what MAWC adds beyond hedging within the router’s regimes. Las Vegas (a) and (b) are PVDAQ systems 1276 and 1278, two installations at the same location.

Reference	Maine	Las Vegas (a)	Las Vegas (b)	Golden, CO
Uniform average	0.629 (−0.059)	0.623 (−0.115)	0.583 (−0.126)	0.523 (−0.065)
Constant convex weights	0.593 (−0.023)	0.598 (−0.090)	0.559 (−0.103)	0.608 (−0.150)
Linear stacking	0.581 (−0.011)	0.581 (−0.073)	0.541 (−0.085)	0.593 (−0.135)
Per-regime convex weights	0.577 (−0.007)	0.526 (−0.018)	0.480 (−0.023)	0.505 (−0.047)
MAWC	0.570	0.508	0.456	0.458
Regime oracle	0.590 (−0.020)	0.508 (−0.000) ^†^	0.462 (−0.005) ^†^	0.375 (+0.083)

**Table 6:** Ablations: (**a**) Two hard selectors with MAWC’s feature map—the argmax of MAWC’s own weights, and an ensemble of five selectors trained by cross-entropy to pick the operator with the smallest error in each development cell, so that the hard alternative is trained for selection rather than read off a network trained to blend. (**b**) Feature groups removed one at a time; each entry compares a five-network ensemble without the group against a five-network ensemble with all features under the same seeds, as the mean change over the four held-out sites, with the number of sites at which the paired interval excludes zero. Las Vegas (a) and (b) are PVDAQ systems 1276 and 1278, two installations at the same location.

(a) Convex Weights Versus Hard Selection					
Site	MAWC	Argmax	Change	Trained Selector	Change
Maine	0.570	0.653	+14.5%	0.656	+15.0%
Las Vegas (a)	0.508	0.595	+17.2%	0.582	+14.6%
Las Vegas (b)	0.456	0.543	+19.1%	0.548	+20.0%
Golden, CO	0.458	0.579	+26.5%	0.616	+34.4%
**(b) Feature Groups**					
**Removed**	**Mean Change**		**Sites with a Significant Change**		
Gap geometry	+4.9%		4 worse		
Channel identity	+1.8%		3 worse		
Calendar	+1.2%		1 worse		
Cross-channel	+0.7%		1 worse		
Long gap × station dark	+0.6%		1 worse		
Periodic availability	+0.3%		1 worse		

**Table 7:** Learning curve: held-out RMSE of a single combiner against the number of development cells it is trained on, mean ± standard deviation over five seeds, each seed drawing its own subsample and initialization. The variation between training sizes is of the same order as the variation between seeds. Las Vegas (a) and (b) are PVDAQ systems 1276 and 1278, two installations at the same location.

Training Cells	Maine	Las Vegas (a)	Las Vegas (b)	Golden, CO
2990	0.601 ± 0.006	0.536 ± 0.011	0.489 ± 0.012	0.518 ± 0.031
5981	0.582 ± 0.007	0.526 ± 0.010	0.479 ± 0.009	0.496 ± 0.011
14,954	0.579 ± 0.002	0.515 ± 0.006	0.468 ± 0.008	0.477 ± 0.008
29,909	0.578 ± 0.002	0.517 ± 0.006	0.468 ± 0.007	0.471 ± 0.011
74,773	0.575 ± 0.004	0.510 ± 0.003	0.465 ± 0.007	0.468 ± 0.010
149,546	0.577 ± 0.001	0.511 ± 0.002	0.463 ± 0.002	0.459 ± 0.009
299,093	0.577 ± 0.003	0.514 ± 0.002	0.466 ± 0.003	0.467 ± 0.006

**Table 8:** Held-out RMSE by regime. The best operator is the pool member with the lowest error in that regime at that site, known only in hindsight; the last column is the operator receiving MAWC’s largest mean weight in the regime. Las Vegas (a) and (b) are PVDAQ systems 1276 and 1278, two installations at the same location.

Site	Regime	Share	FAIR	MAWC	Best Operator	MAWC’s Top Weight
Maine	Transient	0.45	0.462	0.446	0.458 (SAITS)	Linear interp. (0.88)
	Single-sensor	0.23	0.703	0.600	0.616 (SAITS)	Linear interp. (0.33)
	Blackout	0.32	0.762	0.687	0.762 (persistence)	persistence (0.29)
Las Vegas (a)	Transient	0.43	0.385	0.379	0.385 (Linear interp.)	Linear interp. (0.87)
	Single-sensor	0.22	0.520	0.451	0.512 (SAITS)	Linear interp. (0.30)
	Llackout	0.35	0.626	0.608	0.626 (persistence)	Linear interp. (0.32)
Las Vegas (b)	Transient	0.43	0.375	0.370	0.375 (Linear interp.)	Linear interp. (0.87)
	Single-sensor	0.22	0.562	0.479	0.562 (MICE)	Linear interp. (0.30)
	Blackout	0.35	0.505	0.473	0.505 (persistence)	Linear interp. (0.31)
Golden, CO	Transient	0.27	0.159	0.149	0.159 (Linear interp.)	Linear interp. (0.91)
	Single-sensor	0.19	0.208	0.371	0.208 (MICE)	persistence (0.29)
	Blackout	0.55	0.650	0.564	0.645 (resid. cond. Gauss.)	persistence (0.30)

**Table 9:** Sensitivity of the MAWC–FAIR comparison. Each row rebuilds the pool outputs and retrains the ten-network ensemble on the development sites under a changed configuration. Entries are MAWC minus FAIR in held-out RMSE, with ^†^ marking a paired 95% interval that includes zero; the last column counts the held-out sites at which MAWC lies significantly below the regime oracle. The blackout- and sensor-dominated rows set the dominant regime’s share to 0.06 and the other two to 0.015. Las Vegas (a) and (b) are PVDAQ systems 1276 and 1278, two installations at the same location.

Configuration	Maine	Las Vegas (a)	Las Vegas (b)	Golden, CO	Below Regime Oracle
Reference, α=0.03	−0.060	−0.023	−0.031	−0.043 ^†^	1 of 4
Reference, 40 realizations	−0.061	−0.032	−0.029	−0.014 ^†^	1 of 4
SAITS retrained, seed 1	−0.060	−0.025	−0.029	−0.054 ^†^	1 of 4
SAITS retrained, seed 2	−0.059	−0.027	−0.031	−0.051 ^†^	1 of 4
Low missingness, α=0.01	−0.082	−0.006 ^†^	+0.010 ^†^	+0.099	0 of 4
High missingness, α=0.06	−0.066	−0.036	−0.035	−0.018 ^†^	3 of 4
Blackout-dominated	−0.077	−0.018	−0.059	−0.012 ^†^	2 of 4
Sensor-fault-dominated	−0.079	−0.055	−0.044	+0.006 ^†^	2 of 4

^†^ interval includes zero.

## Data Availability

All data used in this study are publicly available. The two development records (Alice Springs and Yulara) are from the DKA Solar Centre historical data archive [24], used under terms that require the DKA Solar Centre to be acknowledged as the source. The four held-out records are systems 1239, 1276, 1278 and 1289 of the NREL PVDAQ public datasets [25], distributed through the Open Energy Data Initiative under DOI 10.25984/1846021. The code, the mask seeds, the configuration files, the trained MAWC networks and the per-realization results needed to reproduce every table and figure have been provided to the journal as Supplementary Materials.

## Supplementary Materials

The following supporting information can be downloaded at https://www.mdpi.com/article/10.3390/s26206589/s1, Code S1: Python source code that builds the operator pool and the mixed masks, trains the policies, and generates every table and figure; Data S1: mask seeds, configuration files, trained MAWC networks and per-realization results.

## Appendix A. Natural Missingness and Segment Inflation

**Table A1:** The records’ own missingness and the segment correction. Left: share of cells missing in the full record on a regular time grid, the share of those cells that FAIR’s diagnosis assigns to each regime, and the median length of the containing gap. Right: under the conventional construction of evaluation segments from the index of complete rows, the share of masked cells whose real gap duration exceeds its nominal duration (mean over 20 realizations) and the largest inflation factor observed. Las Vegas (a) and (b) are PVDAQ systems 1276 and 1278, two installations at the same location.

Site	Missing	Transient	Single-Sensor	Blackout	Median Gap (h)	Inflated Cells	Max. Factor
Alice Springs	1.6%	0.08	0.50	0.42	149.4	3.6%	4
Yulara	7.1%	0.39	0.55	0.06	24.8	33.9%	452
Maine	1.0%	0.21	0.40	0.39	12.8	21.9%	5
Las Vegas (a)	1.5%	0.16	0.00	0.84	24.0	3.2%	17
Las Vegas (b)	0.7%	0.76	0.00	0.24	0.2	1.6%	8
Golden, CO	2.5%	0.02	0.00	0.98	24.0	0.7%	1
Hong Kong	3.4%	0.12	0.79	0.08	12.9	–	–

## Appendix B. Design Choices Re-Derived from the Development Sites

Two choices in this paper were inherited from the authors’ unpublished preliminary comparison, which included one of our held-out sites: the pool’s membership and FAIR’s dispatch table. Table A2 re-derives both from the development sites alone, using the same cached pool outputs as the main results. Each member is the closest estimate at a non-trivial share of cells in at least one regime, so none is redundant for a combiner. The lowest-error member per regime gives the development-only router’s table: linear interpolation for transient loss, MICE for single-sensor faults and SAITS for blackouts. It agrees with FAIR’s table in the first two regimes and differs in the third.

**Table A2:** Development–site evidence for the pool and the dispatch, computed on Alice Springs and Yulara only. For each regime: RMSE of each pool member, and (in parentheses) the share of cells at which it is the closest member. Every member is the closest at a non-trivial share of cells in at least one regime. Bold marks the member with the lowest RMSE in each regime.

Regime	Linear Interp.	Persistence	MICE	Resid. Cond. Gauss.	Cond. Gaussian	SAITS
Transient	**0.351** (0.67)	0.830 (0.11)	0.677 (0.05)	0.817 (0.07)	1.048 (0.04)	0.664 (0.06)
Single-sensor	0.825 (0.23)	0.896 (0.29)	**0.724** (0.11)	0.858 (0.17)	1.068 (0.08)	0.729 (0.11)
Blackout	0.974 (0.28)	0.890 (0.29)	0.882 (0.07)	0.872 (0.20)	1.380 (0.07)	**0.845** (0.09)
All cells	**0.742** (0.43)	0.867 (0.22)	0.768 (0.07)	0.847 (0.14)	1.184 (0.06)	0.749 (0.08)

## Appendix C. Training Variability of a Single Combiner

**Table A3:** Training variability of a single combiner. Each of 20 seeds trains one network on the same development cells; the columns give the mean, standard deviation and range of its held-out RMSE, and the share of seeds at which it is significantly better than FAIR and at which it lies below the regime oracle. The last two columns are two ensembles of ten networks each, formed from disjoint seed sets. Las Vegas (a) and (b) are PVDAQ systems 1276 and 1278, two installations at the same location.

Site	Single: Mean ± SD	Range	Sig. Better than FAIR	Below Regime Oracle	Ensemble A	Ensemble B
Maine	0.577 ± 0.002	0.573–0.581	100%	100%	0.570	0.571
Las Vegas (a)	0.517 ± 0.004	0.510–0.523	20%	0%	0.508	0.510
Las Vegas (b)	0.468 ± 0.003	0.461–0.474	30%	5%	0.456	0.461
Golden, CO	0.472 ± 0.010	0.457–0.490	0%	0%	0.459	0.459

## Appendix D. Supplementary Metrics

**Table A4:** Supplementary metrics. RMSE with its 95% bootstrap interval over realizations; MAE; RMSE on the PV power channel alone and on daytime PV cells (true power above 1% of the site maximum); and the absolute relative error of the PV energy recovered over the missing cells, a downstream quantity in physical units. Las Vegas (a) and (b) are PVDAQ systems 1276 and 1278, two installations at the same location.

Site	Policy	RMSE	95% CI	MAE	PV RMSE	Daytime PV RMSE	PV Energy Error
Maine	Pre-committed	0.717	[0.694, 0.740]	0.406	0.731	1.097	22.8%
	FAIR	0.630	[0.619, 0.642]	0.382	0.407	0.677	7.9%
	Dev.-only router	0.631	[0.614, 0.651]	0.387	0.461	0.761	13.0%
	MAWC	0.570	[0.560, 0.581]	0.340	0.390	0.661	7.0%
Las Vegas (a)	Pre-committed	0.764	[0.716, 0.813]	0.335	1.014	1.163	18.9%
	FAIR	0.531	[0.465, 0.602]	0.184	0.283	0.390	2.6%
	Dev.-only router	0.546	[0.482, 0.614]	0.208	0.323	0.440	4.7%
	MAWC	0.508	[0.442, 0.579]	0.177	0.278	0.384	2.9%
Las Vegas (b)	Pre-committed	0.742	[0.699, 0.794]	0.327	1.015	1.132	14.8%
	FAIR	0.488	[0.427, 0.557]	0.179	0.278	0.384	2.4%
	Dev.-only router	0.501	[0.442, 0.569]	0.203	0.342	0.470	5.4%
	MAWC	0.456	[0.393, 0.529]	0.168	0.278	0.386	3.1%
Golden, CO	Pre-committed	0.982	[0.770, 1.209]	0.621	1.114	1.198	79.7%
	FAIR	0.502	[0.425, 0.589]	0.274	0.537	0.790	36.1%
	Dev.-only router	0.573	[0.499, 0.646]	0.329	0.626	0.903	30.8%
	MAWC	0.458	[0.407, 0.508]	0.264	0.485	0.723	24.1%

## Appendix E. Further Analyses

**Table A5:** Further analyses, single combiners unless stated. (**a**) Five-seed means of MAWC with the mask-only feature map, with a Huber loss, and with a feature map that additionally reads observed context through the pool’s own outputs (each member’s estimate, the pool’s spread, and each member’s deviation from the pool median); ^†^ marks a paired interval against the mask-only model that includes zero. (**b**) A combiner trained on one held-out site and applied to all four. Las Vegas (a) and (b) are PVDAQ systems 1276 and 1278, two installations at the same location.

(a) Loss and Feature Variants				
Variant	Maine	Las Vegas (a)	Las Vegas (b)	Golden, CO
Mask-only (squared error)	0.576	0.515	0.464	0.469
Huber loss	0.583	0.523	0.472	0.464 ^†^
Mask + observed context	0.596	0.529 ^†^	0.486	0.564
**(b) Trained on One Site, Tested on Each**				
**Trained on**	**Maine**	**Las Vegas (a)**	**Las Vegas (b)**	**Golden, CO**
Maine	0.525	0.524	0.485	0.516
Las Vegas (a)	0.570	0.451	0.434	0.462
Las Vegas (b)	0.570	0.476	0.402	0.449
Golden, CO	0.647	0.592	0.546	0.311
development sites	0.578	0.513	0.461	0.474
